# Group-2 innate lymphoid cell-dependent regulation of tissue neutrophil migration by alternatively activated macrophage-secreted Ear11

**DOI:** 10.1038/s41385-020-0298-2

**Published:** 2020-05-26

**Authors:** Veera Panova, Mayuri Gogoi, Noe Rodriguez-Rodriguez, Meera Sivasubramaniam, Helen E. Jolin, Morgan W. D. Heycock, Jennifer A. Walker, Batika M. J. Rana, Lesley F. Drynan, Michael Hodskinson, Richard Pannell, Gareth King, Mark Wing, Andrew J. Easton, Caroline A. Oedekoven, David G. Kent, Padraic G. Fallon, Jillian L. Barlow, Andrew N. J. McKenzie

**Affiliations:** 1 Medical Research Council, Laboratory of Molecular Biology, Cambridge, Cambridgeshire CB2 0QH UK; 2 School of Life Sciences, University of Warwick, Coventry, CV4 7AL UK; 3 Stem Cell Institute, Clifford-Allbutt Building, Hills Road, Cambridge, CB2 0AH UK; 4 Trinity Biomedical Sciences Institute, Trinity College Dublin, Dublin, Ireland; 5 Present Address: The Francis Crick Institute, London, NW1 1AT UK; 6 Present Address: Department of Biology, University of York, Wentworth Way, York, YO10 5DD UK

## Abstract

Type-2 immunity is characterised by interleukin (IL)-4, IL-5 and IL-13, eosinophilia, mucus production, IgE, and alternatively activated macrophages (AAM). However, despite the lack of neutrophil chemoattractants such as CXCL1, neutrophils, a feature of type-1 immunity, are observed in type-2 responses. Consequently, alternative mechanisms must exist to ensure that neutrophils can contribute to type-2 immune reactions without escalation of deleterious inflammation. We now demonstrate that type-2 immune-associated neutrophil infiltration is regulated by the mouse RNase A homologue, eosinophil-associated ribonuclease 11 (Ear11), which is secreted by AAM downstream of IL-25-stimulated ILC2. Transgenic overexpression of Ear11 resulted in tissue neutrophilia, whereas Ear11-deficient mice have fewer resting tissue neutrophils, whilst other type-2 immune responses are not impaired. Notably, administration of recombinant mouse Ear11 increases neutrophil motility and recruitment. Thus, Ear11 helps maintain tissue neutrophils at homoeostasis and during type-2 reactions when chemokine-producing classically activated macrophages are infrequently elicited.

## Introduction

The cytokines interleukin (IL)-25 and IL-33 are released rapidly by epithelial cells in the lungs^1,2^ and intestine^3,4^ in response to type-2 immunity-inducing stimuli, such as allergens and helminthic parasites. These molecules drive type-2 immune responses, promoting the secretion of IL-4, IL-5, IL-9, and IL-13 initially from group 2 innate lymphoid cells (ILC2s),^5–7^ and subsequently from T helper 2 (Th2) cells.^8,9^ This promotes eosinophilia, mucus production, smooth muscle contraction, IgE class switching in B cells, and alternatively activated macrophages (AAM). These responses are required for the robust and timely elimination of parasitic helminth infections in the gut, promoting repair and immune homoeostasis, but also for mediating inappropriate immune activation to allergens. By contrast highly pro-inflammatory pathogen-elicited type-1 immune responses, driven by IFN-γ and TLR ligation, activate classically activated macrophages (CAM) leading to their production of inflammatory cytokines, nitric oxide and chemokines such as CXCL1 that recruit neutrophils to sites of microbial infection. However, even during immune homoeostasis and induced type-2 responses neutrophils are observed without the development of overt type-1 immunity. This suggests that alternative mechanisms exist to recruit and maintain neutrophils in the absence of type-1 stimulation.

During type-2 immunity macrophages respond to IL-4 and IL-13, derived from ILC2s in the innate phase of the response, and express a characteristic alternatively activated profile with high levels of Arginase 1, Relmα, Ym-1, and Ym-2.^10^ AAMs have been implicated in responses associated with repair and metabolic functions, but also in immune pathology. Interestingly, these AAMs also help maintain immune homoeostasis by counteracting inflammatory immune activation characterised by CAM.^10^ Thus, stimulation of AAM or CAM pathways contribute to maintaining the balance of repair and anti-microbial immunity, and potential immune-mediated pathology.

Although many studies have demonstrated a role for AAMs in tissue remodelling and wound repair, by promoting proliferation and collagen production in fibroblasts,^11–13^ there is evidence that AAMs are also able to perform other functions, including activation of T helper cell subsets and pathogen/host cell killing.^14^ However, since AAMs have been shown to be only weakly phagocytic,^15^ the exact mechanism by which they induce pathogen killing is unclear, and it has been suggested that they also recruit neutrophils to specific tissue sites.^10,16^ Indeed, it has been reported that AAMs and neutrophils collaborate to kill *Strongyloides stercoralis* in a complement-dependent manner.^17^ In addition, lung macrophages taken from mice 45 days post *Nippostrongylus* *brasiliensis* infection, and transferred to naive animals, promoted parasite clearance from the worm-infected recipients via a mechanism dependent on ‘N2’ neutrophils (characterised by *Arg1*, *Chi3l3*, and *Retnla* expression).^18^ Neutrophil accumulation, promoted by the chitinases Ym1 and Ym2, has also been reported to contribute to *N. brasiliensis*-induced lung injury.^19^ However, the pathways that govern the presence of neutrophils at sites of type-2 inflammation are understudied, and though a number of factors have been proposed, including the chemokine CCL2,^20^ IL-5,^21^ IL-17A, and Ym1,^19^ their mechanism of action, in some cases, remains to be determined.

We observed that intranasal IL-25 treatment, in addition to inducing eosinophilic inflammation also resulted in a concurrent neutrophil infiltration, which coincided with an increase in expression of the mouse RNaseA paralogue, *eosinophil-associated ribonuclease 11* (*Ear11*). Murine eosinophil-associated ribonucleases (Ear) molecules are paralogues of human eosinophil cationic protein (ECP) and eosinophil-derived neurotoxin (EDN), and are associated with asthma.^22–24^ Assays have suggested diverse roles including chemotactic,^25^ bactericidal,^26^ anti-viral,^27,28^, and anti-helminthic^29^ activities in vitro. Notably, transgenic overexpression of Ear11 in mice resulted in augmented neutrophil development in the bone marrow, and neutrophilic infiltrates in the tissues. Furthermore, naive Ear11-deficient mice had fewer neutrophils in the spleen and lungs, and this specific deficit remained following allergen and worm challenge, while other type-2 immune responses were not impaired. IL-25-dependent expression of *Ear11* was restricted to AAM, was induced by IL-4 and IL-13 treatment, and was ILC2-dependent, with ILC2-deficient mice (*Rora*^*flox/flox*^*Il7raCre*) also showing decreased neutrophilia following IL-25 administration. Mechanistically, administration of recombinant mouse Ear11 (rmEar11) to wild-type mice rapidly recruited neutrophils to the peritoneal cavity. Our data support a novel in vivo role for Ear11 in maintaining tissue neutrophils during homoeostasis and type-2 reactions when differentiation of classical neutrophil chemoattractant-producing macrophages would not normally be promoted.

## Results

### IL-25 induces concomitant tissue eosinophil and neutrophil infiltration in the lungs

**Fig. 1:**
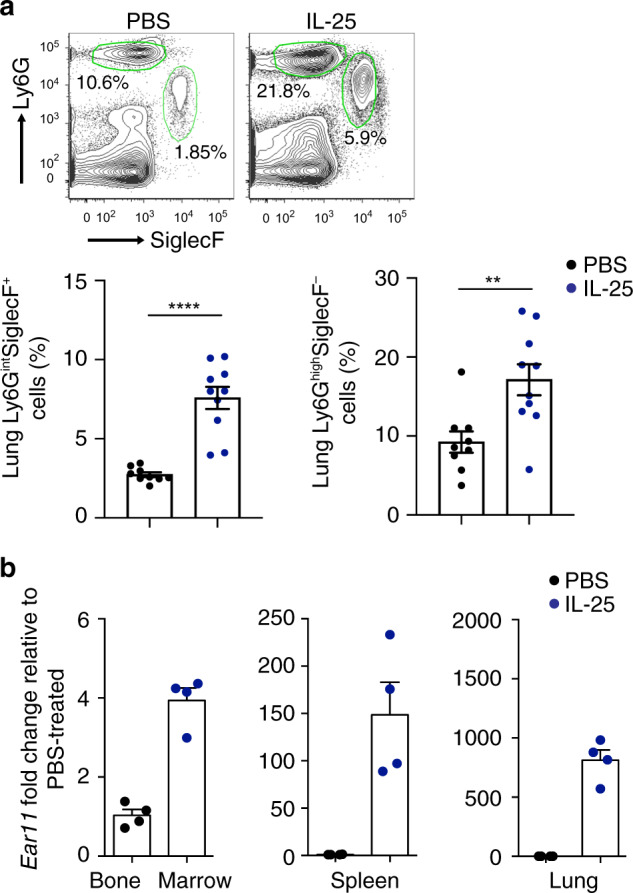
*Ear11* expression is associated with the induction of the type-2 response by IL-25. IL-25 was given intranasally on three consecutive days and lung tissue was analysed. **a** Flow cytometric analysis of eosinophil (Ly6G^int^SiglecF^+^) and neutrophil (Ly6G^high^SiglecF^−^) infiltration. PBS used as a control. Percentage of cells taken from LiveCD45^+^CD11c^−^ gate. Data pooled from two independent experiments (*n* = 4–5). **b** qPCR determination of *Ear11* in bone marrow, spleen, and lung after three doses of IL-25. Data representative of two independent experiments (*n* = 4).

Although IL-25 and IL-33 are primarily potent initiators of eosinophilia,^30,31^ we and others have also observed IL-25-induced neutrophil infiltration in the lungs of mice administered with IL-25 intranasally (Fig. 1a).^30,32^ Time-course analysis also showed a rapid influx of neutrophils in response to IL-33 stimulation (Supplementary Fig. 1a), similar to neutrophil infiltration reported previously in the peritoneal cavity.^33^ Since there is no report of IL-25 receptor expression on neutrophils we performed microarray analysis to study gene expression data from lung tissues exposed to IL-25 and IL-33 to identify potential candidate molecules that could underlie neutrophil infiltration. IL-25 and IL-33 both induced robust upregulation of a number of known type-2 associated molecules, including *Clca3*, *Muc5ac*, *Chi3l3*, *Chi3l4*, *Itln1*, and the chemokines *Ccl11*, and *Ccl17*.^34^ In addition, a number of genes with less well-known type-2 function were also upregulated. One of the most highly upregulated genes was *Ear11*,^34^ a homologue of RNase A, and a paralogue of ECP and EDN. Human EDN and ECP are induced by type-2 immunity^22–24^ and have been reported to possess chemotactic activities in vitro.^25^ Furthermore, Ear11 has been proposed to act in vitro as a chemoattractant for macrophages.^35^ Although mouse *Ear11* expression has been reported in lung alveolar macrophages in models of allergic asthma and following the administration of IL-25 or IL-33, little is known about the functional roles of Ear11 in vivo. To investigate the spatial expression of *Ear11* following the induction of type-2 immune responses, tissues were harvested after intranasal administration of either IL-25 or IL-33. *Ear11* transcription, determined using quantitative reverse-transcription (RT-PCR), was significantly induced in bone marrow, spleen, and lung, and expression was comparable between IL-25- and IL-33-treated tissue (Fig. 1b and Supplementary Fig. 1b).

### Overexpression of Ear11 results in an increased tissue neutrophilia

Given the broad tissue distribution of *Ear11* gene expression we generated transgenic mice with ubiquitous Ear11 overproduction (Supplementary Fig. 2a). Two founder lines were produced and Ear11 overexpression verified (Supplementary Fig. 2b, c). Strikingly, *Ear11*Tg mice from both lines were noticeably smaller than wild-type littermates (Supplementary Fig. 2d), failed to thrive and did not breed. In addition, post mortem analysis revealed abnormalities in tissue morphology, such as small thymi, enlarged hearts, and gall bladders (Supplementary Fig. 2e). In an effort to circumvent the growth abnormalities observed in the *Ear11*Tg mice and more clearly delineate a role for Ear11 production by haematopoietic cells we generated haematopoietic chimaeras with bone marrow harvested from the *Ear11*Tg founders (Supplementary Fig. 2f). Sub-lethally irradiated B6SJL mice (CD45.1) were reconstituted with 100% C57BL/6 (CD45.2) bone marrow (B6SJL:C57BL/6 mice), or *Ear11*Tg (CD45.2) bone marrow (B6SJL:*Ear11*Tg mice).

**Fig. 2:**
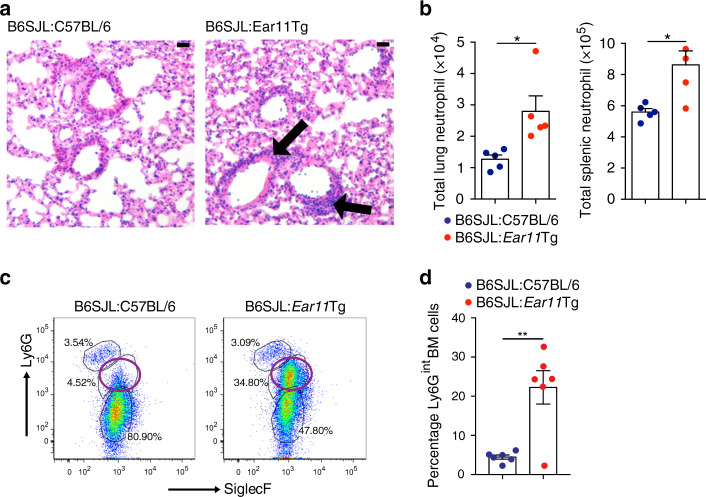
Transgenic overexpression of *Ear11* (*Rnase2a*) causes neutrophil infiltration. **a** Inflammation (black arrows) in the lungs of bone marrow chimaeras was analysed in H&E stained, paraffin-embedded sections (×20 magnification, 20 μm scale bar). **b** Neutrophil numbers assessed by flow cytometry in the lung and spleen of bone marrow chimaeras. **c**, **d** Flow cytometry plots and graphed data showing analysis of SiglecF and Ly6G expression in bone marrow from chimaeras. Plots are gated on LiveCD45.2^+^CD45.1^−^CD11b^+^CD19^−^ cells. Open purple circle denotes a previously uncharacterised Ly6G^int^ bone marrow population shown in the graph (right). Wild-type B6SJL mice received C57BL/6 or *Ear11*Tg bone marrow (BM). Data representative of two independent experiments, *n* = 4–7.

B6SJL:*Ear11*Tg chimaeric mice displayed elevated *Ear11* expression in multiple tissues arising from the transferred bone marrow, and failed to gain weight in the 6 weeks post bone marrow cell reconstitution, as compared with B6SJL:C57BL/6 controls (Supplementary Fig 2g, h). Notably, this correlated with neutrophil infiltration in the lung and spleen (Fig. 2a, b), whereas other inflammatory cells, including T cells and eosinophils, were unchanged (Supplementary Fig. 2i–k). Furthermore, although bone marrow eosinophils (CD45.2^+^CD11b^+^CD19^−^SSC^high^Ly6G^low^SiglecF^+^) and neutrophils (CD45.2^+^CD11b^+^CD19^−^SSC^high^Ly6G^high^SiglecF^-^) were identified but unchanged in the B6SJL:C57BL/6, an unexpected population of Ly6G^int^SiglecF^lo^ cells was observed in the bone marrow of the B6SJL:*Ear11*Tg chimaeras (Fig. 2c, d). This was not due to dysfunctional myeloid development, as assessed by colony forming unit analysis (Supplementary Fig. 2l). Taken together these data indicate an unexpected role for haematopoietic cell-derived Ear11 in regulating neutrophilia in vivo.

### *Ear11* is produced by monocytes, macrophages, and DCs following type-2 provocation in vivo

To examine *Ear11*-dependent pathways and its function in neutrophil homoeostasis and recruitment, an *Ear11* reporter mouse strain was generated by replacing the coding region of the *Ear11* gene with a cassette encoding the mCherry fluorescent protein (Supplementary Fig. 3a). The integration of mCherry created a null *Ear11* allele with breeding to homozygosity producing an *Ear11*-deficient mouse (*Ear11*^*Ch/Ch*^) (Supplementary Fig. 3b). We established the absence of *Ear11* transcripts (Supplementary Fig. 3c, d), and corroborated Ear11 protein deficiency in *Ear11*^*Ch/Ch*^ mice compared with wild-type controls, using antibodies raised against rmEar11 (Supplementary Fig. 4a–e). Pilot studies in naive and type-2 challenged mice did not show any gross difference in *Ear11*mCherry expression between *Ear11*^*+/Ch*^ mice and *Ear11*^*Ch/Ch*^ mice (data not shown), thus although we cannot completely rule out that absence of Ear11 may alter cell expression patterns the available data do not suggest any abnormality.

**Fig. 3:**
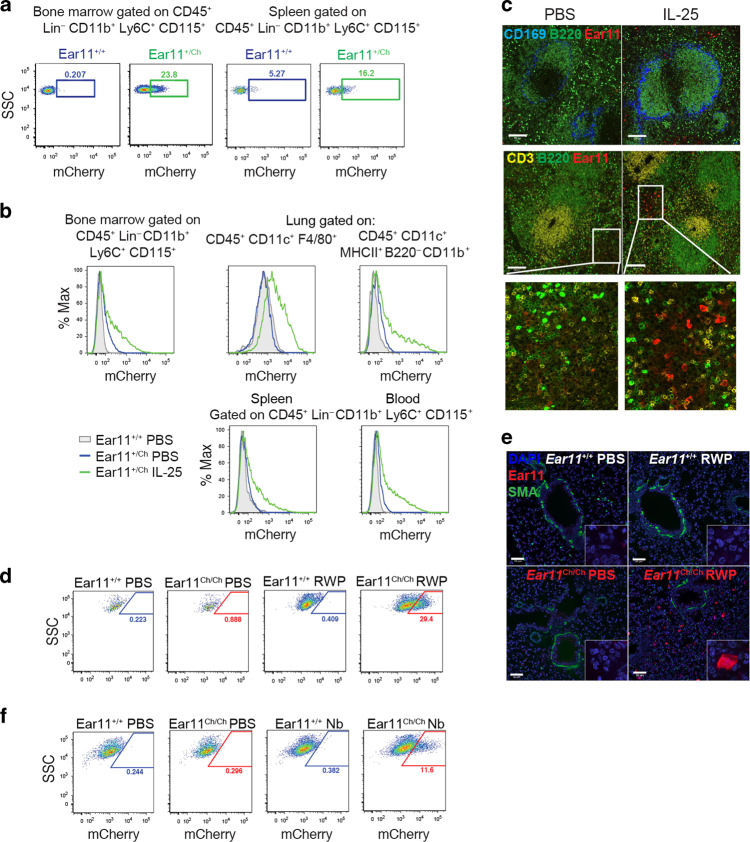
*Ear11*mCherry is expressed in monocytes, macrophages, and DCs during a type-2 response. **a** Flow cytometric analysis of *Ear11*mCherry expression in naive *Ear11*^*+/+*^ and *Ear11*^*+/Ch*^ and **b** mice treated intraperitoneally (bone marrow, spleen, and blood) or intranasally (lung) as indicated (see Supplementary Fig. 5a–d for gating). Representative of 2–3 independent experiments (with 3–4 mice per experiment). **c** Confocal microscopy of spleen sections from *Ear11*^*+/Ch*^ mice treated intraperitoneally as shown (2 independent experiments, *n* = 4). Top row shows the marginal (blue) and B cell zones (green). Middle row depicts T cell (yellow) and B cell (green) zones labelling the white pulp of the spleen (bottom row shows enlargement of *Ear11*mCherry expression in PBS and cytokine-stimulated spleen). ×20 magnification, 100 μm scale bar. **d** Flow cytometric analysis of CD45^+^CD11c^+^F4/80^+^ alveolar macrophages and **e** confocal microscopy of lung taken from *Ear11*^*+/+*^ and *Ear11*^*Ch/Ch*^ mice treated intranasally with PBS or RWP. **e** Airways and blood vessels stained with smooth muscle actin (SMA, green); all nuclei were counterstained with DAPI (blue). Two independent experiments, *n* = 5–8; ×20 magnification with ×63 inset, 50 μm scale bar. **f** *Ear11*mCherry expression in CD45^+^CD11c^+^F4/80^+^ alveolar macrophages on day 32 post infection with *N. brasiliensis* by flow cytometry (data representative of two independent experiments, *n* = 4–7).

In naive *Ear11*mCherry reporter mice, or mice treated with PBS, *Ear11*mCherry expression was detected in CD45^+^ Lin (CD3 CD4 CD8 CD19 Gr-1)^–^ CD11b^+^ Ly6C^+^ CD115^+^ monocytes in bone marrow and spleen, and at lower levels in blood monocytes and lung dendritic cells (Fig. 3a, b and Supplementary Fig. 5a–d). *Ear11*^*+/Ch*^ mice were then treated intraperitoneally or intranasally with PBS or IL-25 to induce a type-2 immune response. IL-25 facilitated a significant increase in *Ear11*mCherry expression in multiple tissues including bone marrow, lung, spleen and blood (Fig. 3b and Supplementary Fig. 5a–d). *Ear11*mCherry^+^ cells in the bone marrow, spleen and blood conformed to a monocyte phenotype, being CD45^+^ Lin (CD3 CD4 CD8 CD19 Gr-1)^–^ CD11b^+^ Ly6C^+^ CD115^+^ (Fig. 3b and Supplementary Fig. 5a–d). In the lung, *Ear11*mCherry expression was observed in CD45^+^CD11c^+^F4/80^+^ alveolar macrophages and in a population of CD45^+^CD11c^+^CD11b^+^MHCII^high^ dendritic cells (Fig. 3b and Supplementary Fig. 5d). Confocal microscopy of frozen spleen sections indicated *Ear11*mCherry^+^ cells in small clusters that localised to the splenic red pulp, and not to the T, B, or marginal zones (Fig. 3c).

To investigate the expression of Ear11 during type-1 and type-2 immune responses *Ear11*mCherry reporter mice were challenged with a number of physiologically relevant immune polarising stimuli. Ragweed pollen (RWP) allergen, when administered intranasally to reporter mice, induced *Ear11*mCherry in alveolar macrophages (Fig. 3d), localised to the sub-mucosa, away from the basement membrane of the large airways (Fig. 3e). In addition, *N. brasiliensis* infection or intranasal *Alternaria alternata* extract challenge induced *Ear11*mCherry expression in lung alveolar macrophages and dendritic cells, respectively (Fig. 3f, Supplementary Fig. 5e, f). By contrast, infection with pneumonia virus of mice (PVM), an enveloped ssRNA—virus that infects lung tissue, and is closely related to human respiratory syncytial virus, failed to induce *Ear11*mCherry^+^ cells (Supplementary Fig. 5g). Similarly, *Ear11*mCherry^+^ cells were not induced in mice treated intranasally with lipopolysaccharide (LPS), a potent inducer of type-1 immunity (Supplementary Fig. 5h).

Together, these data indicate that *Ear11* expression is upregulated during type-2 immunity, but not type-1 immune responses, and is restricted to the myeloid lineage.

### IL-4, IL-13, and ILC2 are required for Ear11 expression by alternatively activated macrophages

**Fig. 4:**
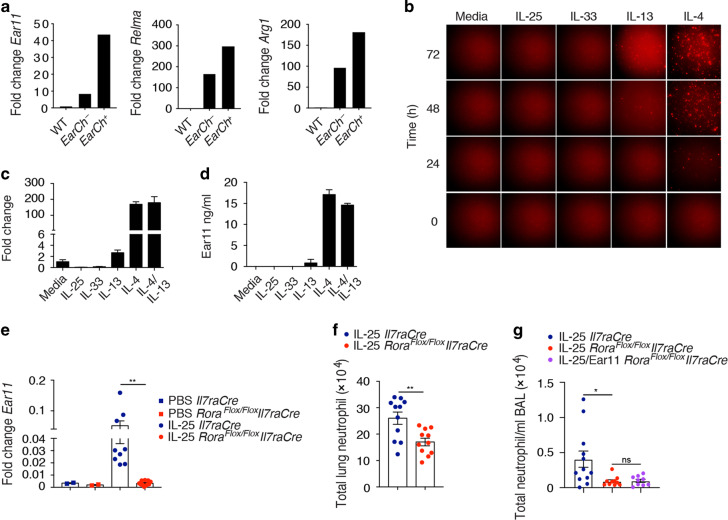
*Ear11*mCherry is expressed preferentially by alternatively activated macrophages. **a** Expression of *Ear11*, *Retnla* (*Relma*), and *Arg1* by alveolar Ear11-negative or Ear11-positive macrophages sorted from *Ear11*^*+/Ch*^ mice treated with intranasal IL-25. Assessed by qPCR. **b** *Ear11*^*+/Ch*^ expression by peritoneal macrophages (CD45^+^CD11b^+^F4/80^+^) cultured in a time-course in either media alone or media supplemented with rmIL-25, rmIL-33, rmIL-13, or rmIL-4 at 10 ng/ml. Cells were imaged every 24 h using a Nikon HCA fluorescence microscope. **c** *Ear11* expression by peritoneal macrophages cultured as indicated (in **b**) for 72 h, assessed by qPCR. Cells from 20 pooled mice, two wells/condition. **d** Ear11 protein concentration in cultures (as in **b**) assessed by ELISA. Data representative of two independent experiments. **e** *Ear11* expression in sorted alveolar macrophages and **f**, **g** total lung neutrophil numbers as assessed by flow cytometry, following two consecutive intranasal doses of IL-25 or IL-25 and Ear11. Data pooled from two independent experiments (*n* = 4–6 per experiment).

The association of Ear11 expression with myeloid cells in type-2 immunity specifically raised the possibility that Ear11 may represent a product of AAMs that arises in response to type-2 cytokine stimulation. Relmα and Arginase 1 are markers of AAM.^36^ Both IL-25 and IL-33 induced Relmα and Arginase 1 expression by lung alveolar macrophages (Supplementary Fig. 6a). IL-25-induced *Ear11*mCherry-positive and -negative alveolar macrophages were isolated and assessed for markers of alternative activation. The *Ear11*mCherry^+^ macrophages were highly restricted to the *Retnla* (*Relma*)-positive, *Arg1*-positive AAM population (Fig. 4a). Similarly, IL-33 induced *Ear11*mCherry^+^ macrophages also showed higher expression of AAM signature genes (Supplementary Fig. 6b, c).

To understand whether IL-25 could directly induce *Ear11* expression, or if this was mediated by cytokines downstream of IL-25, such as IL-4 and IL-13, peritoneal macrophages were purified from naive *Ear11*^*+/Ch*^ mice and cultured with media or the recombinant cytokines IL-25, IL-33, IL-13, and IL-4. IL-25 and IL-33 did not induce Ear11, whilst only a modest induction was caused by IL-13, as assessed by *Ear11*mCherry production (Fig. 4b), the detection of *Ear11* mRNA (Fig. 4c), and Ear11 protein expression (Fig. 4d). By contrast, treatment with IL-4 rapidly upregulated Ear11 (Fig. 4b–d). Ear11 expression by macrophages was not induced by culture with IL-5, IFN-γ, or TLR 1–9 agonists (data not shown).

Since the induction of Ear11 occurs within 24 h of IL-25 treatment,^34^ we assessed whether IL-4 and IL-13-producing ILC2 were required for Ear11 expression and IL-25-induced neutrophil infiltration.^5^ ILC2-deficient (*Rora*^*f/f*^*Il7raCre*) and *Il7raCre* control mice,^8^ were treated intranasally with two consecutive doses of IL-25 and 24 h later lung CD45^+^CD11c^+^SiglecF^+^F4/80^+^ macrophages were sorted and tested for *Ear11* expression by qPCR. Whilst IL-25 administration resulted in highly elevated levels of *Ear11* in macrophages purified from *Il7raCre* control mice, there was a near absence of Ear11 expression in macrophages derived from the ILC2-deficient mice (Fig. 4e). Furthermore, the absence of ILC2 also resulted in reduced neutrophil infiltration as compared with ILC2-sufficient controls upon IL-25 challenge, without affecting homoeostatic neutrophil numbers (Fig. 4f, Supplementary Fig. 6d). Interestingly, complementation of IL-25 challenge with rmEar11 was not sufficient to induce neutrophil recruitment in the lung of ILC2-deficient mice (Fig. 4g).

These results demonstrate that the inducer cytokine IL-25 does not directly induce Ear11 in AAMs, but instead induces IL-4 and IL-13. This likely occurs via an ILC2-dependent mechanism. In turn, IL-4/IL-13-induced AAM upregulate Ear11 production and inducing IL-25-dependent lung neutrophilia.

### Ear11 is required to maintain neutrophil homoeostasis in tissues

**Fig. 5:**
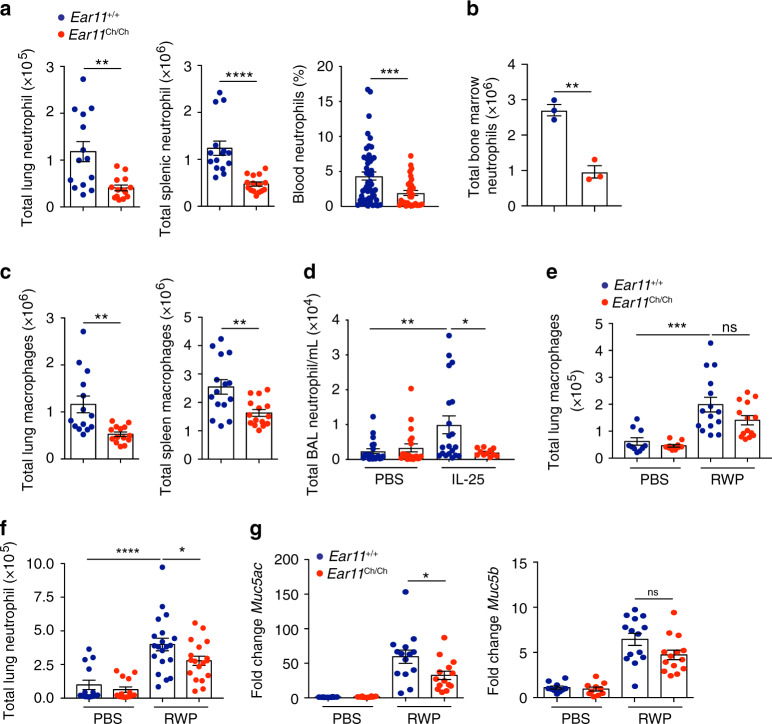
Ear11 helps maintain both naive and type-2 dependent tissue neutrophilia. Flow cytometric analysis of total neutrophil numbers in **a** the lung and spleen, (data pooled from 3 independent experiments, *n* = 3–6 per experiment), blood (data pooled from three independent experiments, *n* = 10–20 per experiment), and **b** bone marrow (representative of two independent experiments, *n* = 6) of naive *Ear11*^*+/+*^ and *Ear11*^*Ch/Ch*^ mice. **c** Total number of lung and spleen macrophages in naive *Ear11*^*+/+*^ and *Ear11*^*Ch/Ch*^ mice. **d** Total number of lung neutrophils in mice treated intranasally with either PBS or IL-25. **e** Total number of lung macrophages in mice treated intranasally with PBS or RWP. **f** Total number of lung neutrophils in mice treated intranasally with either PBS or RWP. **g** Quantitative PCR analysis of *Muc5ac* and *Muc5b* in lung tissue from **e**. **c**–**g** Data representative of two independent experiments, *n* = 5–10 per experiment.

Complementing the previous observations, we found that naive *Ear11*^*Ch/Ch*^ mice had reduced numbers of neutrophils and macrophages in the lung, spleen, blood, and bone marrow in contrast to the increased neutrophilia observed in the *Ear11*Tg mice (Fig. 5a–c). In line with neutrophil reduction, we observed decreased numbers of bone marrow promyelocytes and granulocyte–monocyte progenitors (as previously defined^37^), suggesting a potential role of Ear11 in neutrophil development (Supplementary Fig. 6e). CD4 T cells, CD8 T cells, B cells, ILC2, eosinophils, mast cells, basophils, common myeloid progenitors (CMPs) populations were all normal (data not shown).

Previous studies suggesting an association between Ear11 and lung allergy imply a potential role in type-2 inflammation.^38^ Therefore, wild-type and Ear11*-*deficient mice were next treated intranasally with IL-25 for two consecutive days and 24 h after the last treatment tissues were analysed. In contrast to wild-type mice, *Ear11*^*Ch/Ch*^ mice did not show increased neutrophil infiltration in the bronchoalveolar lavage (BAL) (Fig. 5d). In addition, wild-type mice and *Ear11*^*Ch/Ch*^ were treated with RWP extract for 5 consecutive days before analysis of the tissues 24 h after the last treatment. As expected, RWP treatment increased the numbers of macrophages in wild-type mice, and although the absence of Ear11 did not alter the numbers of macrophages significantly, we did observe fewer neutrophils in the lungs of the *Ear11*^*Ch/Ch*^ mice (Fig. 5e, f). Recruitment of eosinophils to the lung in both IL-25 and RWP challenges was completely independent of Ear11 (data not shown). In addition, *Muc5ac* (but not *Muc5b*) expression in the lungs in response to RWP, assessed as an indicator of mucus production, was decreased in the absence of Ear11 (Fig. 5g).

In vitro assays have led to the proposal that human RNases have anti-helminthic properties.^29^ To investigate the potential in vivo role of Ear11 in gut-induced type-2 immunity to parasitic helminth infection, wild-type and *Ear11*^*Ch/Ch*^ mice were challenged with *N. brasiliensis* larvae. Ear11-deficiency did not alter worm infectivity, cellular infiltration including neutrophils, or cytokine production as compared with wild-type controls in an acute infection model (Supplementary Fig. 7a–c). Furthermore, in a *N. brasiliensis*-induced model of type-2 immune response resolution, in which lung tissues were analysed 32 days post infection, the numbers of macrophages and eosinophils were normal in Ear11-deficient mice. However, neutrophils were found to be reduced in number in *Ear11*^*Ch/Ch*^ mice (Supplementary Fig. 7d).

We hypothesised that other members of the *Ear* gene family may be upregulated during type-2 inflammation and be partially compensating functionally for the loss of *Ear11* (e.g. normal worm clearance and recruitment to some extent of neutrophils). The RNase genes are located in two regions on mouse chromosome 14 (Supplementary Fig. 8a). We compared gene expression levels of known members of the *Ear* gene family (*Ang*, *Ear1*, *Ear2*, *Ear5*, *Ear6*, *Ear10*, and *Ear14*) in the lungs of wild-type and *Ear11*^*Ch/Ch*^ mice (data not shown). Notably, *Ang*, a gene closely related to *Ear11*, was found to be more highly expressed in the lungs of *Ear11*^*Ch/Ch*^ mice as compared with lung samples from wild-type mice (Supplementary Fig. 8b). These data demonstrate that the expression of *Angiogenin* increases in the absence of *Ear11* raising the possibility that *Ear11* and *Ang* genes may have redundant roles in inducing aspects of type-2 immunity.

Together these results indicate that Ear11 contributes to the homoeostasis of neutrophil numbers in normal tissues and helps regulate neutrophil recruitment in response to type-2 immunity-inducing agents. However, despite being highly upregulated by IL-25, RWP and *N. brasiliensis*, in vivo, we were unable to demonstrate an essential role for Ear11 in type-2 inflammation.

### Recombinant Ear11 provokes macrophage and neutrophil recruitment to the peritoneum

**Fig. 6:**
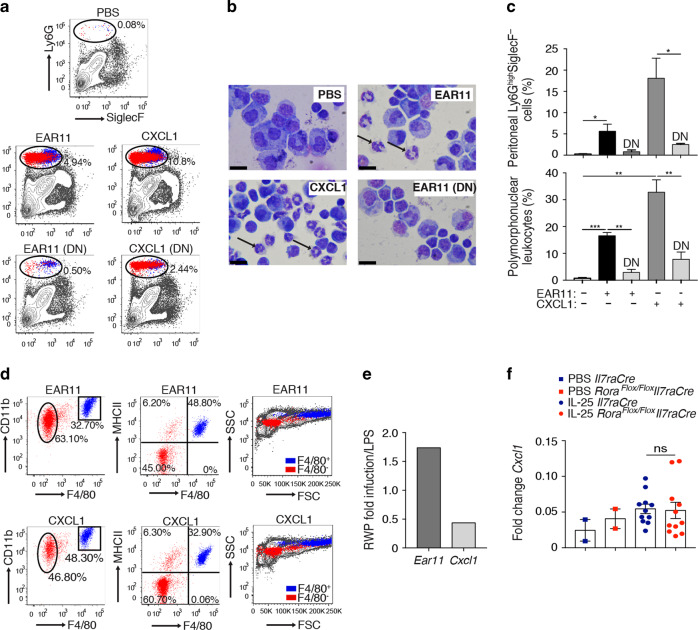
Intraperitoneal administration of rmEar11 induces neutrophil and macrophage recruitment. **a** Flow cytometric analysis of peritoneal lavage cells from naive BALB/c mice treated (native or denatured (DN) protein) as shown (dot plots gated on LiveCD45^+^ cells), and **b** cytospin and Giemsa staining (representative images at ×100 magnification, 20 μm scale bar). **c** Quantification of Ly6G^+^SiglecF^−^ cells from flow cytometry by fixed volume count and of polymorphonuclear leucocytes by differential count (cytospin, bottom panel). Data representative of three independent experiments (*n* = 3–4). **d** Phenotypic analysis of Ly6G^high^SiglecF^−^ cells using CD11b, F4/80, MHCII, and FSC vs. SSC. Cells identified by flow cytometry in the Ly6G^high^SiglecF^−^ gate as ‘macrophage’ coloured blue and as ‘neutrophil’ coloured red. **e** Ratio of *Ear11* and *Cxcl1* gene expression from purified lung macrophages from wild-type mice challenged with ragweed pollen (RWP) or LPS. **f** *Cxcl1* expression in sorted lung macrophages following two consecutive intranasal doses of IL-25. Data pooled from two independent experiments (*n* = 4–5).

To determine the potential mechanism underlying the in vivo role of Ear11 in regulating neutrophils, a single dose of rmEar11 protein (Supplementary Fig. 4a, b), was administered intraperitoneally to mice before harvesting peritoneal lavage (PEL) cells 3 h later. This resulted in the rmEar11-dependent infiltration of CD45^+^Ly6G^high^SiglecF^−^ cells that included neutrophils, as compared with PBS controls (Fig. 6a–c). Additional cell surface markers confirmed that rmEar11 recruited predominantly Ly6G^high^SiglecF^−^CD11b^+^F4/80^−^MHCII^−^SSC^low^ neutrophils and to a lesser extent Ly6G^high^SiglecF^−^CD11b^+^F4/80^+^MHCII^+^SSC^high^ macrophages (Fig. 6d). Furthermore, histological analysis of purified Ear11-induced Ly6G^high^SiglecF^−^ cells confirmed the infiltrate to be composed of ~70% neutrophils and ~30% macrophages (Fig. 6b–d and data not shown). The neutrophil-recruiting activity of rmEar11 was heat labile being lost when the rmEar11 protein was denatured, indicating that potential contamination with heat stable LPS was not responsible for the cellular infiltration (Fig. 6a–c). Interestingly, Ear11 was almost as potent at inducing neutrophil infiltration, as the neutrophil chemoattractant, CXCL1, that is a product characteristic of CAMs (Fig. 6a–d). Together, these results demonstrate that Ear11 provokes neutrophil, and to a lesser degree, macrophage recruitment, which appears to be similar in nature to the infiltrate induced following treatment with the CAM-associated CXCL1. However, Ear11 and CXCL1 were observed to be reciprocally expressed following challenge of mice with either LPS or RWP, with Ear11 characteristic of the type-2 immune response initiated by RWP, and CXCL11 elicited by the type-1 stimulant LPS (Fig. 6e), suggesting that they would each elicit their neutrophilic effect as part of different types of immunological response. Indeed, CXCL1 expression was unchanged in sorted macrophages taken from *Il7raCre* control mice and ILC2-deficient mice treated intranasally with IL-25 (Fig. 6f).

### Ear11 induces chemokinetic movement of neutrophils in the peritoneum

**Fig. 7:**
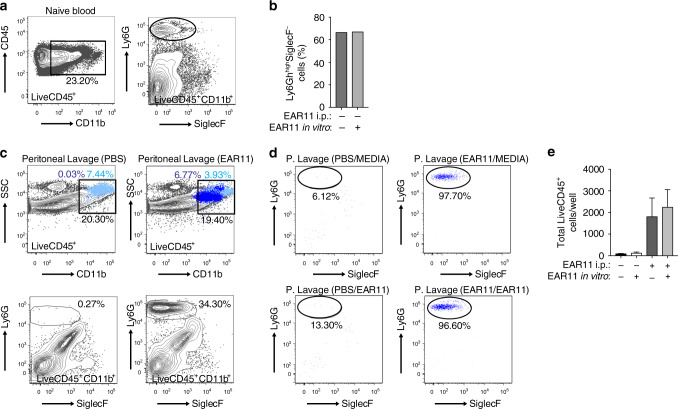
Ear11 mobilises peritoneal neutrophils. **a** Naive LiveCD45^+^CD11b^+^ cells were sorted from blood, and stained with Ly6G and SiglecF to show the presence of neutrophils (Ly6G^high^SiglecF^−^). **b** Sorted LiveCD45^+^CD11b^+^ cells were cultured in an in vitro transwell system with or without 20 ng/ml Ear11 and flow cytometry performed on cells which migrated through the membrane to show the percentage of Ly6G^high^SiglecF^−^ migratory neutrophils. **c** Peritoneal lavage cells sorted from PBS- or Ear11-treated mice as LiveCD45^+^CD11b^+^. Top panel shows light blue cells CD11b^+^Ly6G^int^ and dark blue cells CD11b^+^Ly6G^high^. Bottom panel shows neutrophils gated as Ly6G^high^SiglecF^−^ within LiveCD45^+^CD11b^+^ gate. **d** Sorted cells were cultured as in **b**. **e** Total number LiveCD45^+^ migratory neutrophils collected following Ear11 treatment. Data are representative of two independent experiments.

To directly assess the ability of Ear11 to induce chemotaxis or chemokinesis of cells,^39^ within the Ly6G^high^SiglecF^−^ cell population, in vitro transwell migration assays were performed. To avoid cellular activation through Ly6G ligation during cell purification, the anti-Ly6G antibody staining was omitted and the total macrophage- and neutrophil-containing CD45^+^CD11b^+^ cell population was FACS purified from blood (Fig. 7a) and the migration of these previously unstimulated cells, through a transwell membrane, assessed over a 2-h period in response to Ear11. Although migration was detected this was not restricted to neutrophils, but included CD45^+^CD11b^+^Ly6G^–^ cells, and was not augmented by the presence of Ear11 in the bottom well (Fig. 7b). These data suggest that Ear11 is not a chemotactic factor but is likely to be chemokinetic, i.e. it promotes the general non-directional migration of cells.^39^

To further address this possibility, we purified the total intraperitoneal macrophage- and neutrophil-containing CD45^+^CD11b^+^ cell population from mice 3 h after treatment of mice with either PBS or Ear11 (Fig. 7c). Cell migration through a transwell membrane over a 2-h period was determined in response to media alone or Ear11 in the bottom chamber. In the absence of exogenous Ear11 provocation of the mice, very few Ly6G^high^SiglecF^–^ cells were present in the CD45^+^CD11b^+^ cell fraction purified from PBS-treated PEL. Consequently, the in vitro migration assays contained too few cells for meaningful analysis (Fig. 7d, e). However, pre-treatment of mice with Ear11 resulted in the marked increase of CD45^+^CD11b^+^Ly6G^high^SiglecF^–^ cells in the PEL, that were purified for assessment in migration assays. Interestingly, prior treatment of mice with Ear11 resulted in preferential migration of CD45^+^CD11b^+^Ly6G^high^SiglecF^–^ neutrophils through the membrane (Fig. 7d). In contrast, despite the presence of CD45^+^CD11b^+^Ly6G^–^ cells in the CD45^+^CD11b^+^ cell population in the upper chamber these macrophages did not migrate under these conditions (data not shown). Notably, neutrophil migration was not increased with the inclusion of Ear11 in the lower chamber as compared with media alone controls (Fig. 7d, e), further suggesting that a ‘chemokinetic migratory’ neutrophil phenotype was induced in the neutrophils recruited to the peritoneum by in vivo treatment with Ear11.

Taken together the results demonstrate that Ear11 can provoke increased motility in neutrophils, suggesting that AAMs secreting Ear11 can act to mobilise neutrophils.

## Discussion

Type-2 immunity is characterised by a predominant eosinophilia and the appearance of AAMs, and whilst neutrophils are present they are relatively fewer in number. In part this is because AAMs, in contrast to CAMs, do not express chemoattractants such as CXCL1 that typically drive neutrophil recruitment.^40,41^ Our data suggest that Ear11 production predominantly from monocytes, AAMs, and alternatively activated DCs, contributes to maintaining neutrophil numbers in tissues during homoeostasis, and following type-2 immune activation. In the case of type-2 responses this is at least partially dependent on ILC2. Thus, in an IL-25 driven type-2 immune microenvironment an alternate Ear11-mediated mechanism has arisen to ensure that neutrophils can contribute to the immune reaction without the requirement for TLR and IFN-γ stimulated CAM-derived neutrophil chemoattractants and the escalation of an unwanted and counter-productive pro-inflammatory response.

In the mouse, a large family of small ribonucleases (Ear) show notable sequence and structural homology to human EDN and ECP.^42–44^ The secreted Ear proteins are characterised by an N-terminal signal peptide followed by a ribonuclease A domain containing conserved catalytic histidines and a lysine.^45^ Mouse *Ear11* (also known as *Rnase2a*) transcripts are expressed at basal levels in naive tissues, but can be highly upregulated in response to type-2 immune activation.^35^ Indeed, *Ear11* expression has been shown to be elicited in lung alveolar macrophages following the induction of ovalbumin-induced airways inflammation,^38^ and in response to short and long-term mouse models of allergic asthma.^46,47^ Similarly, administration of the type-2 initiator cytokines IL-25 or IL-33 also induced *Ear11* gene expression.^34,35^ Although most members of this RNase A gene family have not been studied extensively, a recent report indicated that Ear11 may function in vitro as a chemoattractant for tissue macrophages independently of TLR2 signalling.^35^

Although macrophages represent a spectrum of phenotypes influenced by local environmental cues and stimuli, distinct polarised subsets have been described.^10^ CAMs arise in response to stimulation via IFN-γ and TLR ligation leading to their production of inflammatory cytokines, nitric oxide, and chemokines such as CXCL1 that recruit neutrophils to sites of microbial infection. By contrast, the type-2 cytokines IL-4 and IL-13 support AAMs that are often considered anti-inflammatory, promoting quiescence and repair, and restricting inflammatory responses characterised by CAMs. This response helps to restore the damage resulting from parasitic worm invasion, but may lead to inappropriate tissue repair and fibrosis in allergic disease. Macrophages exhibiting an AAM phenotype (also known as M2 or M(IL-4)), characterised by the expression of high levels of *Relma* and *Arg1*, have been reported in multiple tissues,^48^ suggesting a broad physiological role, possibly in maintaining immune homoeostasis and preventing unwanted inflammation. Indeed, tissue-resident ILC2s have been shown to produce IL-4 and IL-13 that sustain AAMs for the establishment of inflammatory thresholds in neonate and adult lungs,^49^ and in adipose tissue to protect from obesity.^50^ Such an environment may be maintained through tissue-resident macrophages self-renewing and proliferating in response to IL-4 without additional recruitment.^51^

Using a new *Ear11*^*Ch/+*^ knock-in reporter mouse, we demonstrated *Ear11* expression in vivo across multiple tissues, cell types, and models of immune activation. Ear11 was highly restricted to monocytes (CD11b^+^ Ly6C^+^ CD115^+^), dendritic cells (CD11c^+^MHCII^high^), and macrophages (CD11b^+^F4/80^+^) and was only induced by type-2 cytokines, allergic challenges, and parasitic infection, but not by pneumoviral (PVM) infection or bacterial (LPS) provocation. Although Ear11 expression was induced by IL-25 and IL-33 in vivo, these cytokines did not upregulate Ear11 production directly from macrophages, but instead this required IL-4, in line with a report that IL-4 provoked *Ear11* transcripts from alveolar macrophages.^35^ AAMs are induced in an IL-4Rα1- and STAT6-dependent manner and are characterised by expression of molecules including the mannose receptor, *Ym1*, *Arg1, Relma*, and *Fizz1*.^52–54^ Our in vivo analysis has established that Ear11 expression was limited to Relmα-positive AAMs. This discovery prompts the future investigation of the syntenic human molecules, EDN and ECP, which may have as yet unappreciated roles in AAM biology and indeed these molecules do seem to be expressed at low levels in RNASeq datasets taken from human AAM cultures.^55^

Exploiting several complementary approaches, including Ear11 overexpression in transgenic mice, Ear11 ablation and exogenous Ear11 administration, we demonstrate here that Ear11 is a monocyte- and AAM-secreted regulator of neutrophil homoeostasis and migration. Consequently, Ear11-enhanced neutrophil recruitment to tissues, such as lung and spleen, was compromised in Ear11-deficient mice. Conversely, mice overexpressing Ear11, had increased neutrophil infiltration to peripheral tissues, but not in bone marrow, perhaps reflecting the normal constitutive naive expression of Ear11 here. In addition, when rmEar11 protein was given to mice it provoked a predominantly neutrophilic cell infiltration, and to a lesser extent macrophage recruitment, within 3 h of its administration. However, administration of Ear11 to ILC2-deficient mice was not sufficient to promote recruitment of neutrophils, suggesting that further, yet to be defined factors are also necessary. Taken together, these results demonstrate that Ear11 production by AAMs represents a novel mechanism for neutrophil maintenance and recruitment to sites of type-2 immunity. Though the role of neutrophils in type-2 immune responses remains controversial, it has been shown that an early migration of neutrophils to the site of infection is necessary to control worm burden.^19,56,57^ Our data using alarmin- and allergen-induced type-2 models support these previous observations. However, other studies suggest production of Th2-derived IL-4 and IL-13 later in the type-2 response can also inhibit neutrophilia.^58–60^ Our data suggest that early IL-4 production, from cell types such as ILC2s, may be required for timely neutrophil infiltration. Thus, these apparently contradictory observations may be reconciled by considering a differential involvement of neutrophils in different stages of the type-2 response.^61^

Interestingly, in naive Ear11-deficient mice there were also fewer neutrophils in the tissues and fewer neutrophil progenitors in the bone marrow, suggesting that in the steady state Ear11 helps to maintain homoeostatic numbers of neutrophils. Further studies are required to determine whether this results from effects on development, proliferation, or survival (or a combination of the three). Ear11 may therefore poise neutrophil infiltration in a manner similar to the way that IL-5 and eotaxin regulate eosinophils.^62–64^ The precise pathways required to maintain neutrophils at tissue sites during homoeostasis are not well characterised, but the rate of neutrophil production, storage in and egress from the bone marrow, and survival in, and movement out of the blood are all proposed to play a role in this ‘neutrostat’.^65^ Our results suggest that Ear11 production by monocytes and AAMs contributes to this process. Interestingly, Ear11 is part of a family of RNases and we noted that in the absence of Ear11 the highly related molecules Angiogenin was upregulated, suggesting that compensatory mechanisms may arise between members of this family. Thus, it may be necessary to remove two or more of these genes to fully explore the impact of RNase-regulated neutrophil induction on immune function.

The rapid influx of neutrophils following intraperitoneal injection of rmEar11 clearly demonstrated its capacity to stimulate neutrophil, and to a lesser extent macrophage, recruitment to the peritoneal cavity. Notably, the migratory effect of Ear11 was only marginally less potent than exogenously administered CXCL1. Interestingly, when we harvested cells from the peritoneal lavage of Ear11-provoked mice we found that the neutrophil component was highly migratory in vitro, whilst these assays were unable to detect macrophages migrating in the same time-frame. It is also noteworthy that once activated in vivo the neutrophils did not respond chemotactically to additional Ear11 provided in the assay system, implying that they are responding chemokinetically to Ear11. These assays could only be performed using cells from Ear11-stimulated mice since these populations were absent from the peritoneal lavage in the absence of Ear11 provocation. In vitro studies carried out previously suggested that rmEar11 provided a chemoattractant signal to naive F4/80^+^CD11c^−^ macrophages within the total splenocyte population assayed, with little effect on Ly6G^+^ cells.^35^ However, we now demonstrate that in vivo the main cell target of Ear11-induced migration is neutrophils, with a lesser but still significant contribution to macrophage recruitment. This difference between the two studies may result from the tissue source of the cells under study (e.g. spleen versus peritoneal lavage) and the importance of maintaining tissue and cellular environments using in vivo approaches.

In conclusion, we have demonstrated a novel in vivo role for Ear11, a close relative of human ECP and EDN proteins, in neutrophil recruitment and maintenance in tissues. Consequently, Ear11 produced by IL-4-activated AAMs as part of type-2 immune reactions, is exquisitely placed to support the infiltration of neutrophils in response to immune challenges that fail to elicit CXCL1-producing pro-inflammatory CAM (Supplementary Fig. 9). Our data also raise the possibility that human RNase molecules may have similar roles in humans and contribute to neutrophilic asthma.

## Methods

### Mice

Overexpression of *Ear11* (*Rnase2a*) in all tissues was achieved by microinjection of wild-type *Ear11* cDNA, under the transcriptional control of CAG promoter in the pCAGGS vector (Supplementary Table 1), into F1 fertilised eggs. Two founder *Ear11* transgenic (Tg) lines were identified. *Ear11*Tg mice did not breed and in vitro fertilisation was used to maintain two of the founder lines (mouse identifier 64572 and 71970).

*Ear11*mCherry reporter mice (*Ear11*^Ch/Ch^) mice were generated by replacing exon 2 of *Ear11* with the mCherry fluorescent protein coding sequence followed by a floxed neomycin resistance gene in JM8.6 (C57BL/6) embryonic stem cells by homologous recombination. Embryonic stem cell clones in which a successful targeting event had occurred were identified using 5′ and 3′ probes (Supplementary Table 1) by Southern blot analysis and used to generate *Ear11*^+/*Ch*^ mice. The neomycin gene was removed in the germline by interbreeding *Ear11*^Ch/Ch^ with a Cre-recombinase expressing strain. The line was then also backcrossed onto the BALB/c background for six generations.

For bone marrow transfer experiments B6SJL (CD45.1) mice were sub-lethally irradiated with two doses of 4.5 Gy γ-irradiation. On the same day they received 1–2 × 10^6^ bone marrow cells (fresh or frozen stocks), from either C57BL/6 (CD45.2) or *Ear11*Tg (CD45.2) mice, by intravenous injection. Mice were reconstituted for at least 6 weeks.

*Il7raCre* and *Rora*^*f/f*^*Il7raCre* mice are previously described.^8^ Age- and sex-matched mice at 6 weeks old or above were used in experiments. Mice were housed in specific pathogen-free conditions. All experiments were undertaken with the approval of the UK Home Office.

### Generation of rmEar11 protein and anti-mouse Ear11 antibodies

Recombinant mouse (rm)Ear11 was expressed as a 6xHis fusion protein in Sf9 cells using Bac-to-Bac Baculovirus Expression System (Thermo Fisher Scientific) and purified from cell culture supernatants on a Nickel NTA resin column (GE Healthcare) followed by gel filtration on HiLoad 16/60 Superdex 75 column (GE Healthcare) and then HisTrap HP column (GE Healthcare). Purified protein was dialysed into sterile, endotoxin-free PBS. Endotoxin concentration was quantified at 32 EU/mg using the Limulus/Amebocyte Lysate (LAL) assay according to manufacturer’s instructions (Pierce, ThermoFisher).

A male rat was immunised by sub-cutaneous injection of 100 μg of rmEar11 combined in a 1:1 ratio with TiterMax (Sigma-Aldrich) on day 0, 28, and 32. A single intravenous injection of 50 μg of Ear11 was given on day 84. Monoclonal antibodies were generated by standard protocols.

### Ear11 protein detection

Ear11 levels in supernatants were assessed using a Meso Scale Diagnostics (MSD) assay. MSD standard binding plates (Meso Scale Diagnostics) were coated with polyclonal anti-Ear11 serum (1:2500 dilution), blocked with 5% Blocker A (Meso Scale Diagnostics) and incubated with standards (serial dilution of rmEar11) and/or samples overnight. Detection antibody, biotinylated monoclonal rat anti-mouse Ear11 antibody (clone BC2.12), was added at 2 μg/ml, followed by streptavidin SULFO-TAG (Meso Scale Diagnostics) and 2x Read buffer T (Meso Scale Diagnostics). The electrochemiluminescent signal was read on a Sector S 600 plate reader.

### Mouse challenge protocols

In all instances recombinant proteins, extracts, and LPS were given in endotoxin-free PBS (Sigma-Aldrich) or PBS as a control.

rmIL-25 (2 μg/dose, made in-house, Janssen Pharmaceuticals), rmIL-33 (0.5 μg/dose, Peprotech) or rmEar11 (2 μg/dose) were given on 2 or 3 consecutive days (as indicated) and tissues harvested 24 h after the final dose. rmEar11 (2 μg/dose) or CXCL1 (0.5 μg/ml, R and D Systems; <100 EU/mg protein) were given as a single dose and tissues were harvested at 12 h post injection. rmEar11 and CXCL1 were boiled for 60 min to denature the protein.

RWP (100–200 μg/dose, RWP, *Ambrosia artemisiifolia*, short form, Greer Laboratories) was given intranasally on 5 consecutive days. All tissues were harvested 24 h after the final dose. Alternatively, three doses RWP were administered intranasally over 3 days and lung CD45^+^CD11c^+^F4/80^+^SiglecF^+^ alveolar macrophages purified 24 h after the final dose. Macrophage gene expression was analysed by qPCR.

LPS (1–2 μg/dose, Ultrapure LPS-EB from *Escherichia* *coli* 0111:B4, InvivoGen) was given intranasally on three consecutive days. Analyses were performed 24 h after the final dose. In some experiments, lung CD45^+^CD11c^+^F4/80^+^SiglecF^+^ alveolar macrophages were purified and gene expression analysed by qPCR.

### Mouse Infection models

Mice were inoculated sub-cutaneously with 500 viable third-stage *N. brasiliensis* larvae in endotoxin-free PBS and tissues were harvested on day 4, 7, 10, and 32 post infection.

A single intranasal dose of 50 PFU of PVM (strain J3666, gift from Prof. Easton) was given in PBS or PBS alone. Tissues were analysed at day 2, 5, and 12 post infection.

### Cell preparation and flow cytometry

Single lung cell suspensions were prepared by incubating finely chopped tissue with 720 μg/ml collagenase D (Roche) in RPMI (Invitrogen) at 37 °C for 30 min, passing the tissue through a 70 μm cell strainer, centrifugation through 30% Percoll (GE Healthcare) in RPMI and lysing RBCs. Single spleen, mediastinal, and mesenteric lymph node cell suspensions were prepared by passing the tissue through a 70 μM cell strainer (except when preparing cells for macrophage subpopulation analysis where tissue was treated with collagenase D, as for lung) and lysing RBCs. Single bone marrow and peritoneal cell suspensions were prepared by flushing the femur and tibia, or peritoneum with endotoxin-free PBS and lysing RBCs. Peripheral blood leukocytes were prepared using Whole Blood Lysing Reagent Kit (Beckman Coulter) according to the manufacturer’s instructions.

Single cell suspensions were incubated with anti-CD16/CD32 antibody (Fc block, clone 93) followed by fluorochrome-conjugated antibodies (Biolegend, unless otherwise indicated) against: mouse Arginase 1 (A1exF5), B220 (clone RA3-6B2), CD3 (clone 145-2C11), CD4 (clone H129.19, BD Biosciences), CD8 (clone 53–6.7), CD11b (clone M1/70), CD11c (clone N418), CD19 (clone eBio 1D3), CD45 (clone 30-F11), CD45.1 (clone A20), CD45.2 (clone 104), CD115 (clone AFS98), CD127 (clone SB/199), F4/80 (clone BM8), FcεR1 (clone MAR-1), Gr-1 (RB6–8C5), ICOS (clone 398.4A), Ly6C (clone HK1.4), Ly6G (clone 1A8-Ly6g), MHCII (M5/114.15.2, Biolegend), Relmα (DS8RELM, eBioscience), SiglecF (E50-2440, BD Biosciences). All samples were co-stained with a cell viability dye (Fixable dye eFluor780, Invitrogen). For analysis samples were run on an LSR Fortessa system (BD Biosciences). For cell sorting an iCyt Synergy (70 μm nozzle, Sony Biotechnology) was used.

Unless otherwise stated alveolar macrophages were defined as CD45^+^CD11c^+^F4/80^+^SiglecF^+^, eosinophils as CD45^+^CD11c^−^F4/80^−^CD11b^+^Gr1^int^ SiglecF^+^, neutrophils as CD45^+^CD11c^−^F4/80^−^CD11b^+^Gr1^high^SiglecF^−^, CD4^+^ T cells as CD45^+^ CD3^+^CD4^+^, and innate lymphoid type-2 cells (ILC2s) as CD45^+^Lin^−^(CD3CD4CD8CD19 CD11bCD11cFcεR1) CD127^+^ICOS^+^.

### Immunofluorescence imaging

Lung and FALC were prepared by fixation in 4% paraformaldehyde at 4 °C for 1 h, incubating in 20% sucrose overnight for cryoprotection, and embedding in 15% sucrose/7.5% gelatin diluted in PBS. Overall, 12 μm frozen sections were cut using a Leica CM 3050 S cryostat. Sections were pre-blocked/permeabilized in PBS containing 3% BSA and 0.05% Triton X-100 and stained with primary antibodies anti-mCherry (M11217, Invitrogen), anti-Relmα (500-P214, PeproTech), or anti-actin α-smooth muscle FITC conjugated (1A4, Sigma), followed by secondary antibodies anti-rat IgG Alexa Fluor 568 (A11077, Life Technologies) and anti-rabbit IgG Alexa Fluor 488 (A21206, Life Technologies) for 1 h at the room temperature, or directly conjugated CD3 (clone 145-2C11), B220 (clone RA3-6B2), and CD169 (clone 3D6.112). Image acquisition was performed using either a Zeiss LSM 780 confocal microscope and ZEN 2010 acquisition software or a Nikon HCA fluorescence microscope and NIS-Elements 4.30.01 acquisition software for live cell imaging. Adobe Photoshop 11.0.2 was used to adjust brightness, contrast and colour balance (changes were applied to all images in equal measure).

### In vitro culture of primary cells

Sorted peritoneal macrophages were cultured for 72 h in RPMI media (Gibco) with 10% fetal bovine serum (Labtech), 1% penicillin/streptomycin and 100 μM 2-β-mercaptoethanol (BDH Biochemicals) with or without 10 ng/ml IL-33 (Biolegend), 10 ng/ml IL-25 (Janssen), 10 ng/ml IL-13 (eBioscience), or 10 ng/ml IL-4 (R&D Systems). Cell free supernatants were collected for ELISA and cells were lysed using Trizol LS reagent (Life Technologies) for RNA extraction.

Cells were flushed from the bone marrow through a 70 μm cell strainer and incubated with the following fluorochrome-conjugated antibodies against: CD5 (clone 53–7.3), CD117 (clone 2B8), Sca1 (clone D7), CD34 (clone RAM34), CD16/32 (clone 93), as well as lineage markers as previously described (CD11b, B220, Gr1, TER119, CD19). Fixable viability dye eFluor780 (Invitrogen) was also added. CMP cells were then sorted as LiveLineage^−^CD5^−^CD117^+^Sca1^−^CD16/32^int^CD34^+^ and plated at 100 cells/well in MethoCult^TM^ with GF M3434 media (STEMCELL Technologies). Colony number and type were analysed at day 9 using the STEMvision system with the custom mouse CSC algorithm.

Live CD45^+^CD11b^+^ cells were sorted from peritoneal lavage of PBS- and Ear11-treated mice, or from naive blood and plated on 24 well transwell inserts (PET, 3.0 μm pore, Falcon) at between 2.5 and 3.0 × 10^5^ cells/well. Media alone or 10 ng/ml rmEar11 was added to the bottom well and cells were incubated at 37 °C for 2 h, after which cells that had migrated through the membrane were subject to flow cytometry.

### Reverse-transcription (RT)-PCR and real-time quantitative (q) PCR

RNA was isolated using RNA Bee reagent (Amsbio) or Trizol LS reagent (Life Technologies) according to the manufacturer’s instructions. All RNA samples were treated with DNase I using RNeasy Micro Kit (Qiagen) and reverse-transcribed into complementary DNA using Super-RT enzyme (HT Biotechnology). RT-PCR was performed using KOD Hot Start DNA polymerase (Novagen) and self-designed primers (Table S1). Products were run on a 2% agarose gel and visualised using a Bio-Rad ChemiDoc XRS + System and Image Lab 5 software. Real-time qPCR was performed using SYBR Green mastermix (Applied Biosystems) and self-designed primers or Taqman Universal PCR master mix (Applied Biosystems) and commercially available Taqman gene expression assays (Table S1, Applied Biosystems). Samples were run on the ViiA7 real-time PCR system (Applied Biosystems).

### Histology

Tissue was fixed in 10% formalin overnight and paraffin-embedded. Sections were stained with haematoxylin and eosin. Image acquisition was performed using a Nikon Labophot-2 biological microscope and Camera Control Pro 2 software. Peritoneal lavage cells were subject to cytospin and differential counting.

### Statistical analyses

Results are shown as mean ± SEM. Results were considered significant at *P ≤ 0.05; **P ≤ 0.01; ***P ≤ 0.001; ****P ≤ 0.0001. Statistical analyses were performed using Prism version 6.0f.

## Supplementary information

Supplementary Information
