# Supplementary material for: Group-2 innate lymphoid cell-dependent regulation of tissue neutrophil migration by alternatively activated macrophage-secreted Ear11
Source: Mucosal Immunol. 2020 May 26;14(1):26–37. doi: 10.1038/s41385-020-0298-2 (PMC7790759; doi:10.1038/s41385-020-0298-2)
Supplement: Supplementary file 1 — Supplementary Information [file 41385_2020_298_MOESM1_ESM.pdf]

Supplementary Figure Legends

Supplementary Fig. 1. *Ear11* expression is associated with the induction of the type-2 response by IL-33. IL-33 was given intranasally on 1, 2 or 3 consecutive days and lung tissue was analysed. **a** Flow cytometric analysis of eosinophil ($Ly6G^{int}SiglecF^{+}$) and neutrophil ($Ly6G^{high}SiglecF^{-}$) infiltration. PBS used as a control. Percentage of neutrophils taken from $LiveCD45^{+}CD11c^{-}$ gate. Data pooled from two independent experiments (n = 9-10). **b** qPCR determination of *Ear11* in bone marrow, spleen, and lung after 3 doses of IL-33. Data representative of two independent experiments (n = 4).

Supplementary Fig. 2. Generation of a transgenic mouse to overexpress *Ear11*. **a** Enhancer/promoter elements and *Ear11* transgene. Black triangles; position of genotyping primers. **b** Detection of transgene insertion by PCR, as compared to wildtype littermate control. **c** qPCR analysis of *Ear11* in wildtype and *Ear11*Tg littermate controls. Data representative of two independent founder lines. **d** Body size and weights of *Ear11*Tg mice compared to wildtype littermates, sex-matched controls (n = 11 from 2 founder lines). **e** Representative gross tissue morphology of wildtype and *Ear11*Tg mice; 1 thymus, 2 heart, 3 gall bladder. **f** Haematopoietic chimaera experimental protocol. **g** Percentage weight change of mice receiving C57BL/6 or *Ear11*Tg bone marrow (data from one experiment, n = 16). **h** qPCR analysis of *Ear11* in tissues of chimaeric mice. Eosinophils and $CD4^{+}$ T cells in **i** lung and **j** spleen of chimaeric mice. **k** $CD4^{+}$ T cells and ILC2s in mesenteric lymph nodes. All tissues were taken 6 weeks post-reconstitution. Data representative of 2 similar experiments (n = 5). **l** Analysis of BM colony forming unit (CFU) assays of CMPs taken from wildtype and *Ear11*Tg mice. Total number of colonies (left panel) and colony type (right panel, CFU-M, monocyte, CFU-G, granulocyte, CFU-GM, macrophage/granulocyte, CFU-GEMM, granulocyte/erythroid/monocyte/megakaryocyte) analysed on day 9 of culture. Data are representative of pooled data from three independent experiments (n = 2 mice per experiment).

Supplementary Fig. 3. Generation of *Ear11*Cherry reporter mice. **a** Schematic showing *Ear11* targeting construct and arms of homology to wildtype allele. mCherry insertion replaces exon 2 of *Ear11*. Black bars, probes for Southern blotting; black triangles, position of genotyping primers. **b** Representative Southern blot analysis of *Ear11*mCherry targeted ES cell clones with 3′ probe (top panel) and a representative PCR screen (bottom panel) using

genomic ear DNA from *Ear11*$^{+/+}$, *Ear11*$^{+/Ch}$, and *Ear11*$^{Ch/Ch}$ mice. *Ear11*-deficiency was confirmed in tissues taken 24 hrs after treating mice with three daily intranasal doses of IL-33. **c** RT-PCR and **d** quantitative PCR analysis.

Supplementary Fig. 4. Generation of recombinant mouse (rm)Ear11 and anti-Ear11 antibodies. **a** Schematic showing the plasmid for expression of mouse Ear11 protein. **b** SDS-PAGE Coomassie-stained gel showing; M marker, T total load, L lysate, FT flow through, washes and elution for rmEar11 protein purification. **c** Western blot analysis of rmEar11 using either polyclonal serum or monoclonal biotinylated anti-Ear11 (clone BC2.12) for primary detection. **d** Optimisation of the Ear11 MSD sandwich assay with polyclonal anti-Ear11 antiserum as the capture antibody and biotinylated anti-Ear11 (clone BC2.12) as the detection antibody. **e** Ear11 protein assessed by MSD assay in supernatants taken from macrophages stimulated *in vitro* with 10 ng/ml IL-4 for 72 hrs.

Supplementary Fig. 5. *Ear11*Cherry expression is detected *in vivo* in response to type-2, but not type-1 immunity. Gating strategy for *Ear11*mCherry-expressing $CD45^+CD11b^+Ly6C^+CD115^+$ monocyte populations for **a** bone marrow, **b** spleen, and **c** blood. Defined as $CD45^+Lin^-$(CD3CD4CD8CD19Gr1)$CD11b^+Ly6C^+ CD115^+$. **d** Gating strategy for alveolar macrophages (left panel), defined as $CD45^+CD11c^+F4/80^+$ (also $SiglecF^+$ and $CD11b^-$, not shown), and lung DCs (middle and right panels) defined as $CD45^+CD11c^+MHCII^{high}B220^-CD11b^+$. **e** Flow cytometric analysis of lung DCs (defined as in **d**) and **f** confocal microscopy of lung (20x magnification with 63x inset, 50 μm scale bar) taken from *Ear11*$^{+/+}$ and *Ear11*$^{Ch/Ch}$ mice treated intranasally with PBS or *A. alternata* extract (2 independent experiments, n = 4 – 7 per experiment). SMA; smooth muscle actin. **g** Flow cytometric analysis of *Ear11*mCherry expression in alveolar macrophages from *Ear11*$^{+/+}$ and *Ear11*$^{Ch/Ch}$ mice infected intranasally with PBS or pneumovirus of mice (PVM) (data representative of two experiments, n = 5 – 7 per experiment, day 5 post infection). **h** Flow cytometric analysis of *Ear11*mCherry expression by alveolar macrophages in *Ear11*$^{+/+}$ and *Ear11*$^{Ch/Ch}$ mice dosed intranasally on three consecutive days with PBS or 1-2 μg LPS. Lung tissue taken 24 hrs after final challenge (representative of two independent experiments, n = 4 – 5 per experiment).

Supplementary Fig. 6. IL-25 and IL-33 induce production of Ear11 from alternatively activated macrophages. **a** Flow cytometric analysis of Relmα and Arginase 1 expression by

alveolar macrophages taken from mice treated with two consecutive intranasal doses of IL-25 or IL-33. PBS given as control. **b** *Ear11*$^{+/Ch}$ expression in Relmα^{+} macrophages in frozen sections of fat-associated lymphoid tissue (FALC) from *Ear11*$^{+/Ch}$ mice treated intraperitoneally with IL-33 and assessed by confocal microscopy (upper panel 20x magnification, 20 μm scale bar; lower panel 63x magnification, 10 μm scale bar). **c** Expression of *Ear11*, *Retnla* (*Relma*), and *Arg1* by alveolar Ear11-negative or Ear11-positive macrophages sorted from *Ear11*$^{+/Ch}$ mice treated with intraperitoneal IL-33. Assessed by qPCR. **d** Total lung neutrophils in naïve *Il7raCre* and *Rora*$^{Flox/Flox}$*Il7raCre* mice. **e** Total number of promyelocytes (LiveCD45^{+}CD11b^{+}Ly6G^{int}) and granulocyte-monocyte progenitors (GMPs, LiveCD45^{+}Lineage^{-}CD117^{+}Sca1^{-}CD34^{+}CD16/32^{high}) in the bone marrow of *Ear11*$^{+/+}$ and *Ear11*$^{Ch/Ch}$ mice.

Supplementary Fig. 7. Acute and chronic response to *N. brasiliensis* in *Ear11*-deficient mice. **a** Number of worms per intestine at the times indicated following *N. brasiliensis* infection (ND, not detected). **b** Flow cytometric analysis of indicated lung cells at day 4 post-infection. **c** Quantitative PCR analysis of *Il4*, *Il5*, and *Il13* expression in the lung tissue at day 4 post-infection. All data representative of two independent experiments, n = 6 – 10 per experiment. **d** Indicated lung cell numbers at day 32 post-infection with *N. brasiliensis* (data representative of two independent experiments, n = 4 - 7).

Supplementary Fig. 8. The *Ear11* paralogue, *Angiogenin*, is upregulated in *Ear11*-deficient mice. **a** Schematic of the mouse *Ear* gene loci. *Ear* gene family members and closest relative *Ang* highlighted in red. **b** Quantitative PCR analysis of *Ang* in the lung of *Ear11*$^{+/+}$ and *Ear11*$^{Ch/Ch}$ mice treated with intranasal doses of PBS or RWP (data pooled from two independent experiments, n = 10 – 14).

Supplementary Fig. 9. Schematic representation of the role of Ear11 in maintaining neutrophils at homeostasis and in type-2 immune responses.

Supplementary Figure 1

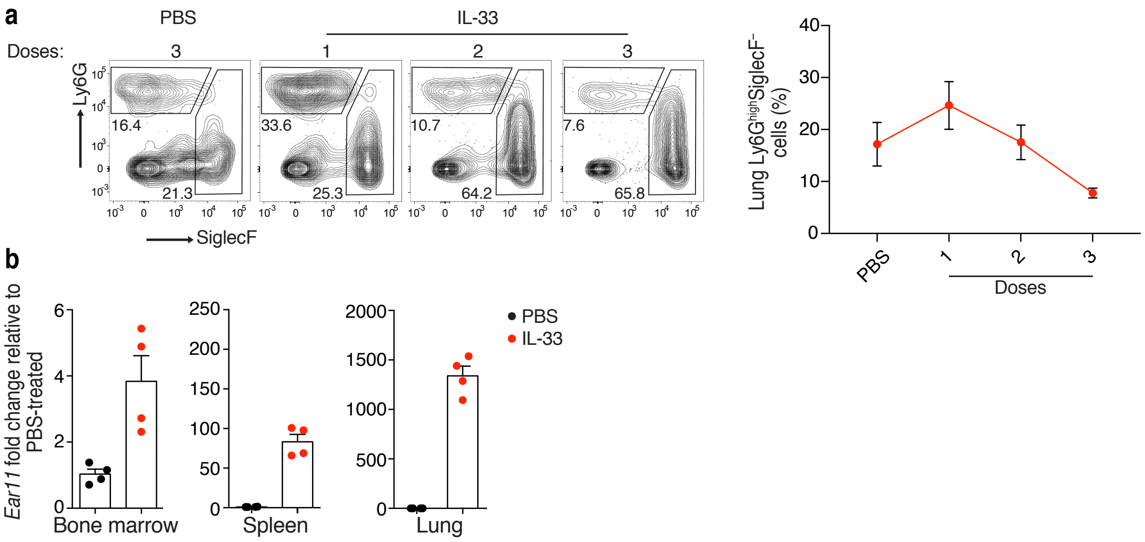

Supplementary Figure 2

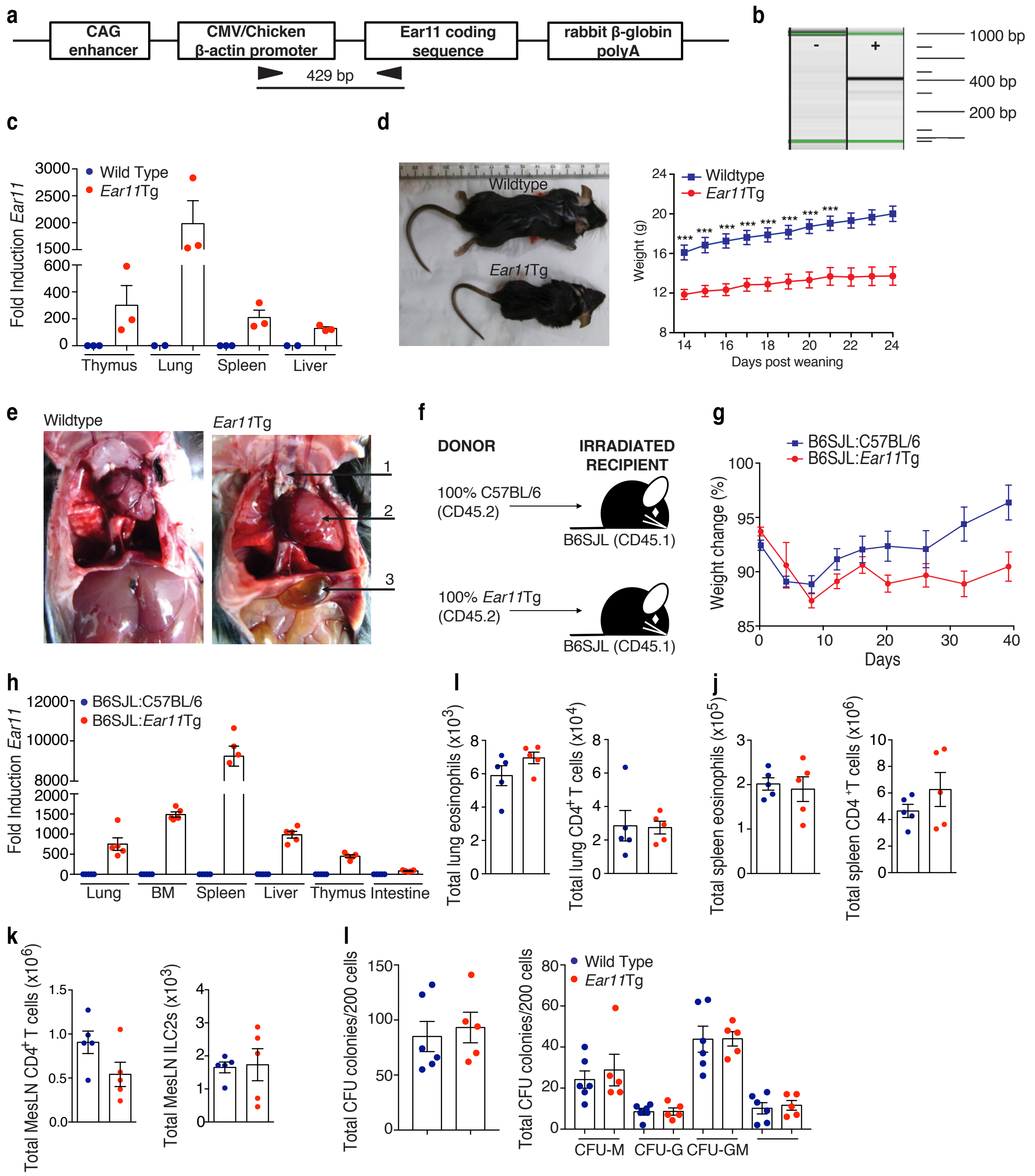

Supplementary Figure 3

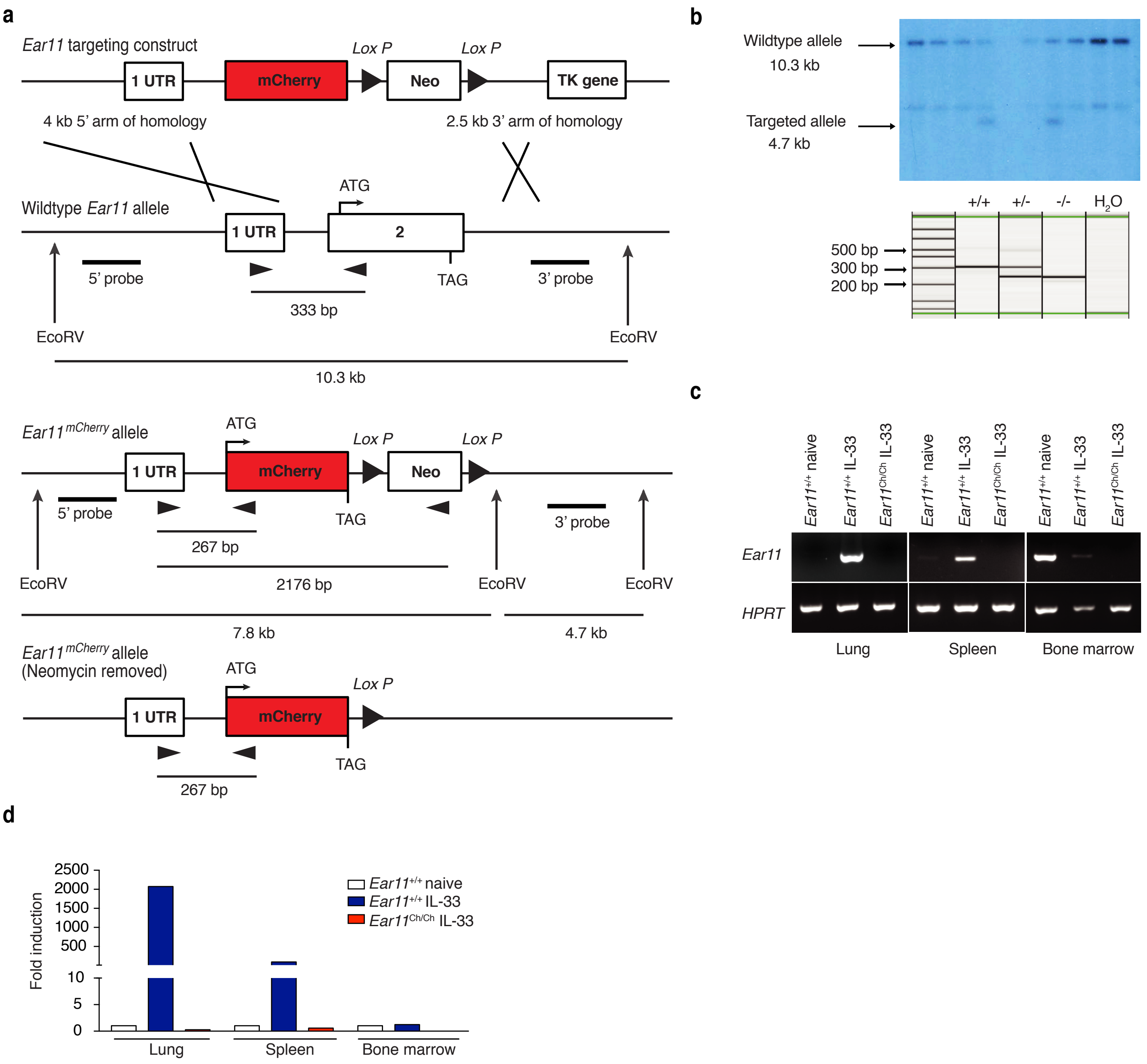

Supplementary Figure 4

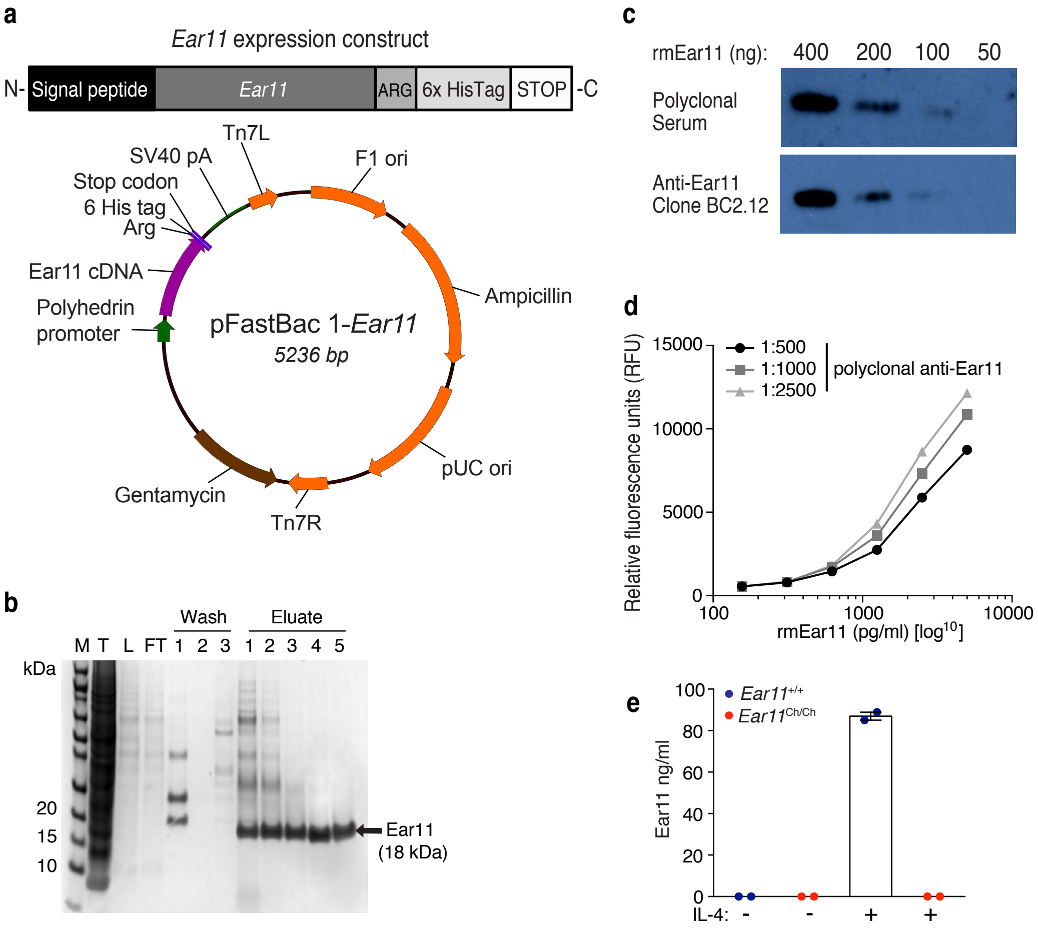

Supplementary Figure 5

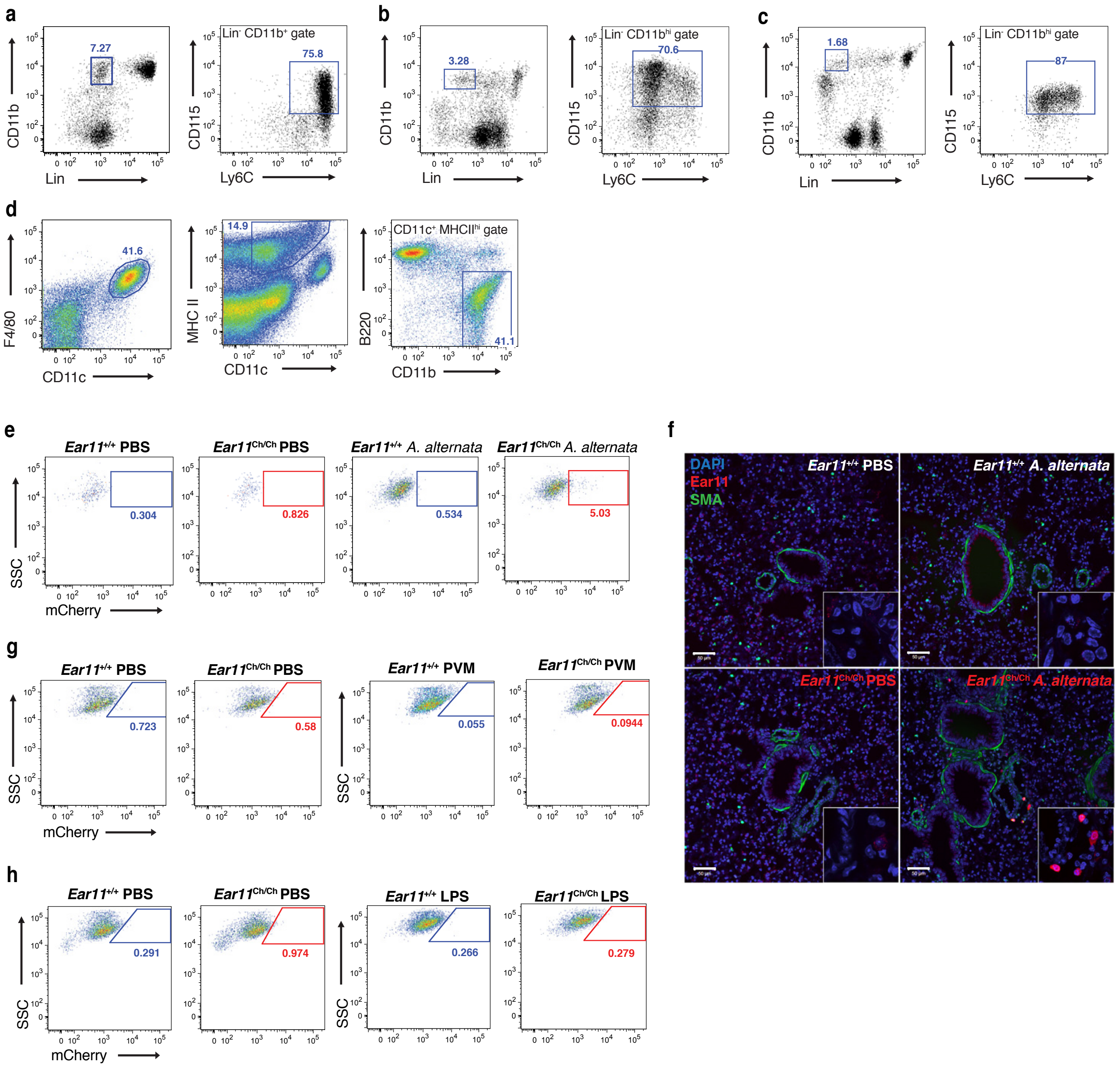

Supplementary Figure 6

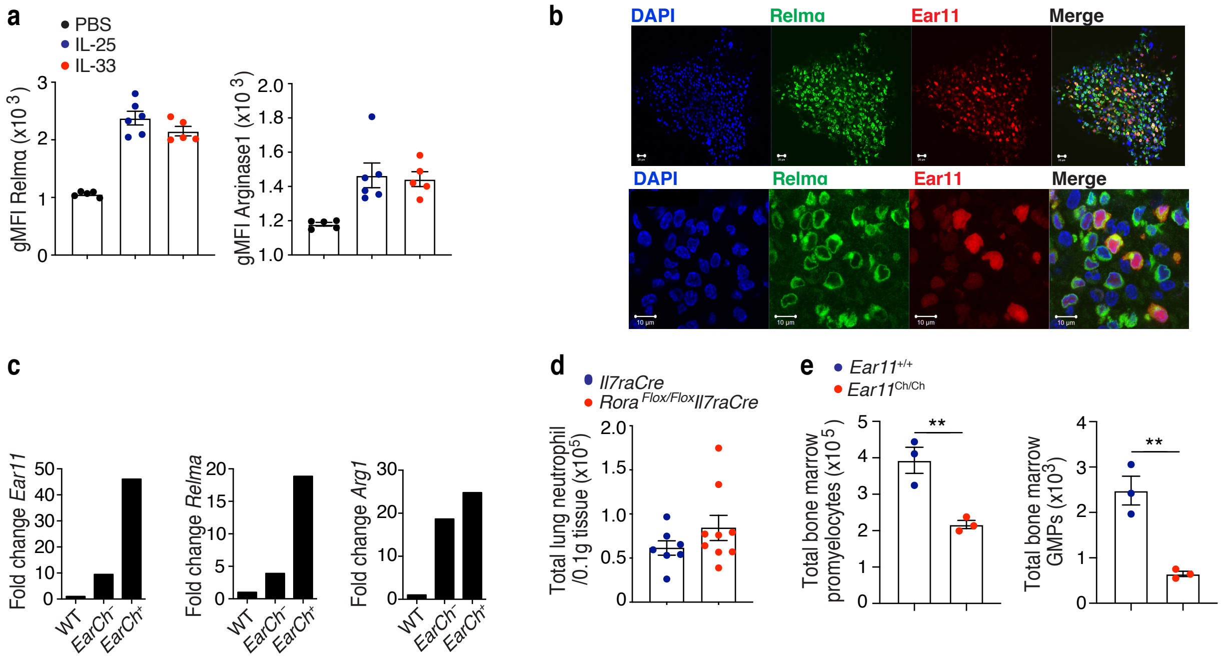

Supplementary Figure 7

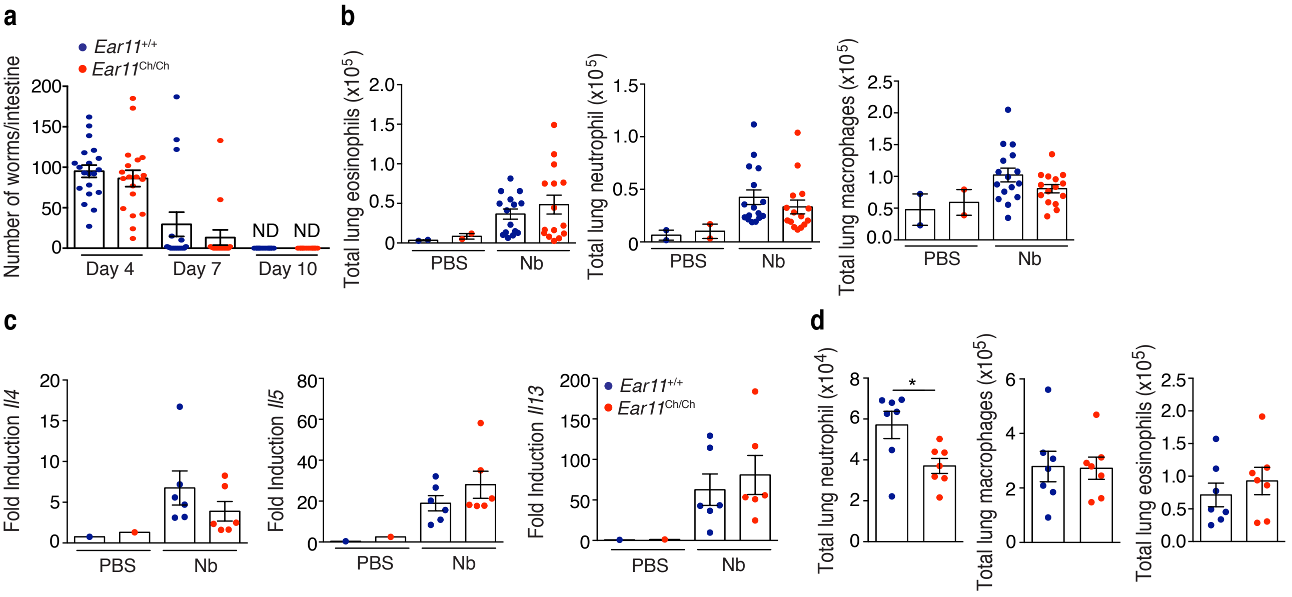

Supplementary Figure 8

a

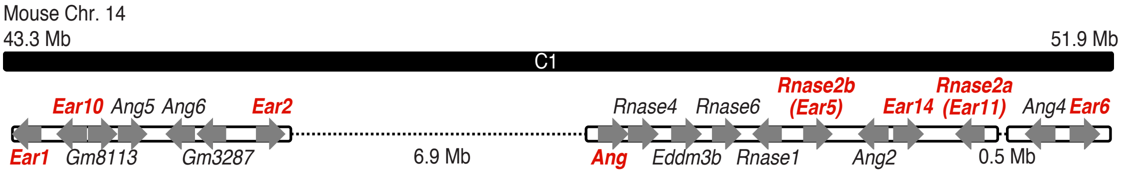

b

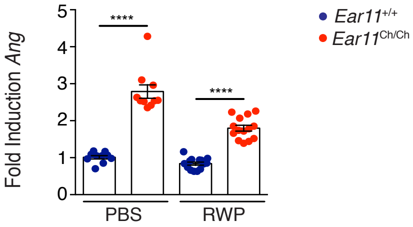

Supplementary Figure 9

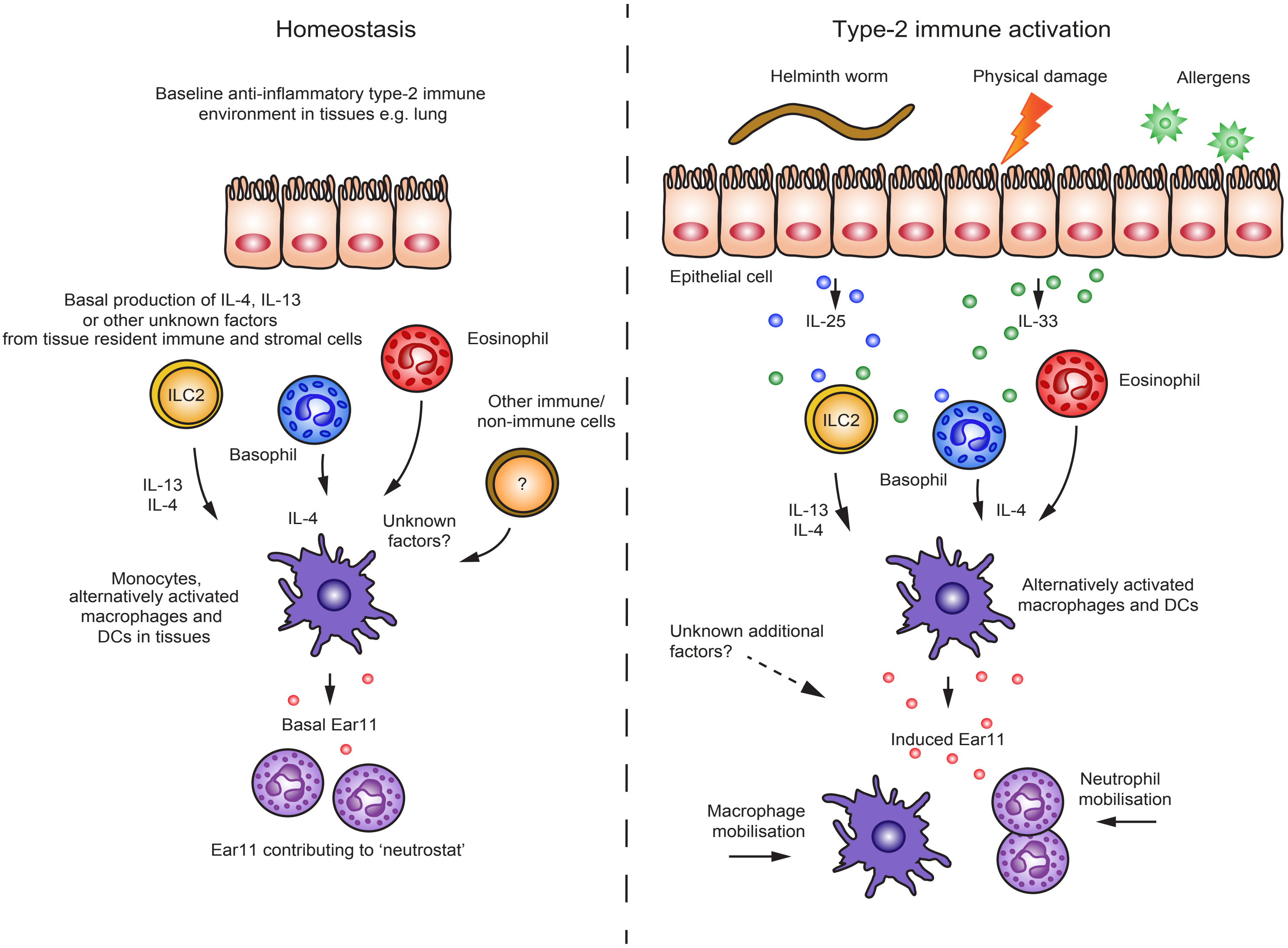

Supplementary Table 1.

Primers for generation of *Ear11*$^{Ch/Ch}$ mouse

Primer use	Primer sequence		Product length bp
	Forward	Reverse	
Genomic *ear11* sequence in 7 kb	TGT AGC TCA GCG GCC GCT CAT TTC TCT AG	GAC CAT CAT GCT AGC TCT AAT CTA TTG CAT	7037
Recombineering - to insert mCherry/Neomycin cassette into *ear11* gene deleting exon 2	AAC AGA GTT AGG GTG GGA ATA GCC GCT TCT CTT CTG TTC TCA CAG GAA ACC GCC ACC ATG GTG AGC AAG GGC GAG GAG G	GCT AAA TTA AAG GGA GTC AGC AAC AGG TTT CAT GAG GAC AGA TGC TGA CAG ATA TCC TAG TGG ATC CCC TCG AGG GAC	2939
Southern 5' probe	AGA CAT CTA TCA GTT GGG AC	TCC ACA GTG ACA CAT AGA TAG	504
Southern 3' probe	ACA CGC TGC AGA GAT ACC TGC	ACA CTT GCA CAG GCA AAG ACA C	520
Genotyping wild type allele	AGT TCC ACG GGA GCC ACA AAG CAG ACT GG	TCG GGG ATA GGC TCT GTT ATA GAT ATG CTG	333
Genotyping mCherry^{+} allele		TGC ACC TTG AAG CGC ATG AAC TCC TTG ATG	267
Genotyping Neomycin presence/absence		AGG TGA GAT GAC AGG AGA TC	2176

Primers for generation of *EAR11*Tg mouse

Primer use	Primer sequence		Product length bp
	Forward	Reverse	
Ear11 cDNA	TAT CGA ATT CAT GGG TCT GGA GCA ACT TGA GTC	TAT CGA ATT CCT AAA ATA TCC CAT CCA AGT GAA C	488
Genotyping	TGC CTT TTA TGG TAA TCG TGC	TCC CAG CAG CAG CAG GAG AC	429

Primers for RT-qPCR

Gene name	Primer sequence		Product length bp
	Forward	Reverse	
HPRT	GTT GGA TAC AGG CCA GAC TTT GTT G	GAG GGT AGG CTG GCC TAT AGG CT	396
Ear11	GTC TGG AGC AAC TTG AGT CTC	GGC AGT AGC CGA TGA ATG AAG	507
Ear1&2	CTT TGC ACC TTT GCT CAT CTG	TAA AGG GAG TAA GTG ACA GG	114
Ear5	ACT TGT CCT AAC GCT TGT CTC	GTC CCG TCT TTG CAA GTC TTG	219
Ear6	TGA CTA TCT AGG CCT GGG AC	GAC TAC TCC CAC GCT ACC TC	133
Ear10	GCT GAG AAC ATA GTA TTG AAG	GTA AGT GAA GAT CTG TTC AGC	115
Ear14	TGA TGC TGT AAT GAG GGT TG	ACC TGT CGT TTG GTA TCT GC	232
Angiogenin	AGC CCA GGC CCG TTG TTC TTG	GAT GTT GCT CTT GTT GCC ATG	222

TaqMan gene expression assays

Gene name	Assay number
GAPDH	4352932E
Ear11	Mm 00519056_s1
Relmα	Mm 00445109_m1
Ym1	Mm 00657889_mH
Arg1	Mm 00475988_m1
Muc5ac	Mm 01276718_m1
Muc5b	Mm 00466391_m1
